# Analysis of Gene expression in soybean (*Glycine max*) roots in response to the root knot nematode *Meloidogyne incognita *using microarrays and KEGG pathways

**DOI:** 10.1186/1471-2164-12-220

**Published:** 2011-05-10

**Authors:** Heba MM Ibrahim, Parsa Hosseini, Nadim W Alkharouf, Ebtissam HA Hussein, Abd El Kader Y Gamal El-Din, Mohammed AM Aly, Benjamin F Matthews

**Affiliations:** 1 United States Department of Agriculture, Plant Sciences Institute, Beltsville, MD 20705, USA; 2 Department of Computer and Information Sciences, Towson University, Towson, MD 21252, USA; 3 Genetics Department, Faculty of Agriculture, Cairo University, Giza, Egypt; 4 Department of Arid land Agriculture, College of Food and Agriculture, UAE University, UAE

## Abstract

**Background:**

Root-knot nematodes are sedentary endoparasites that can infect more than 3000 plant species. Root-knot nematodes cause an estimated $100 billion annual loss worldwide. For successful establishment of the root-knot nematode in its host plant, it causes dramatic morphological and physiological changes in plant cells. The expression of some plant genes is altered by the nematode as it establishes its feeding site.

**Results:**

We examined the expression of soybean (*Glycine max*) genes in galls formed in roots by the root-knot nematode, *Meloidogyne incognita*, 12 days and 10 weeks after infection to understand the effects of infection of roots by *M. incognita*. Gene expression was monitored using the Affymetrix Soybean GeneChip containing 37,500 *G. max *probe sets. Gene expression patterns were integrated with biochemical pathways from the Kyoto Encyclopedia of Genes and Genomes using PAICE software. Genes encoding enzymes involved in carbohydrate and cell wall metabolism, cell cycle control and plant defense were altered.

**Conclusions:**

A number of different soybean genes were identified that were differentially expressed which provided insights into the interaction between *M. incognita *and soybean and into the formation and maintenance of giant cells. Some of these genes may be candidates for broadening plants resistance to root-knot nematode through over-expression or silencing and require further examination.

## Background

Plant parasitic nematodes cause about US $100 billion in crop losses annually [1,2]. Root-knot nematodes (RKN; *Meloidogyne *spp.) are sedentary endoparasites. The most economically important species are *Meloidogyne incognita *and *M. arenaria*. Both are widespread and are considered as major crop pathogens worldwide. The RKN can be easily recognized by the "knots" or "galls" that form where they feed on roots [3,4]. These nematodes cause dramatic morphological and physiological changes in plant cells. Some plant genes are subverted by nematodes to establish feeding cells, and transcripts of several nematode genes were identified during infection [5]. Root-knot nematode damage to soybean (*Glycine max*) can be severe, especially when fields previously planted in cotton are rotated into soybean [6].

The RKN life cycle is complex [for review see: [3-5,7]]. The egg is laid in the soil or in plant tissues. The first stage juvenile develops inside the egg and molts one time to the second-stage juvenile (J2). When the J2 hatches from the egg, it infects the root close to the root tip in the elongation zone and migrates to the vascular tissue, where it establishes a feeding site by injecting esophageal proteins into several plant cells and it recruits host genes to alter the morphology of the host cells. Host cells become binucleate and then undergo multiple rounds of synchronous mitosis without cell division to form a giant cell [3-5,7]. These multinucleate cells can contain more than 100 polyploid nuclei. The cells surrounding the giant cell undergo hypertrophy and hyperplasia to form a root gall [3-5,7,8]. Thus, expression of numerous host genes is modified to produce these extensive changes in the root. The J2 males and females molt three more times to reach maturity. The mature female produces an egg mass in a gelatinous protective sac that is extruded from the female nematode onto the root surface.

Within the nematode's esophageal gland cells, different proteins are produced to help the nematode establish a feeding site. Some of the proteins secreted by the nematode are injected into host cells and cause modification of the plant cells to form giant cells. Other proteins secreted by the nematode may interact with the host's extracellular receptors to influence signal transduction [8].

Similarly, gene expression is altered in the cells that are selected to be the feeding sites of the soybean cyst nematode. Klink *et al*. [9-13] demonstrated that numerous changes in gene expression occur in roots and in syncytial cells in soybean roots infected by either compatible or incompatible populations of soybean cyst nematodes. They used microarrays to study gene expression in laser capture microdissected (LCM) syncytium cells for susceptible and resistant reactions of soybean during infection with soybean cyst nematode (*Heterodera glycines; *SCN) [9,10,12]. Many genes were shown to be up- and down-regulated in both susceptible and resistant responses. Also, they identified many genes that are involved in plant-pathogen interactions, which provided new insights into the interaction between the cyst nematode and its host plant. In another microarray study by Klink *et al. *[12], distinct expression patterns at different developmental stages of the SCN feeding site were detected in gene expression studies of syncytial cells collected by LCM from SCN-susceptible and resistant soybean cultivars. Gene expression patterns at the first stage were found to be similar in both the susceptible and resistant reactions, when the nematode first attempts to establish itself in the host. This stage is called the parasitism phase. The second stage depends on the defense response of the host plant. If the soybean plant exhibits resistance to the parasite, the nematode will fail to establish and will develop very slowly or die. If the plant is not resistant to the nematode, the soybean host and SCN are compatible and the nematode will establish its permanent feeding site. Using microarray analysis Ithal *et al. *[14,15] studied transcript expression in syncytium cells induced by SCN in soybean roots after infection. They reported that several pathways are involved in the induction of syncytia. For example, genes involved in solidifying and lignifying the cell wall of the syncytium were shown to be up-regulated. Interestingly, they also reported down-regulation of the plant defense system, specifically the pathway leading to jasmonic acid. On the other hand, Klink *et al. *[16] examined the response of a resistant cultivar of soybean against SCN by studying gene expression using microarrays. The levels of transcripts of genes encoding enzymes involved in jasmonic acid biosynthesis, phenylpropanoid biosynthesis, suberin biosynthesis, adenosyl-methionine biosynthesis, ethylene biosynthesis from methionine, flavonoid biosynthesis and the methionine salvage pathway were greatly altered during the defense response of soybean to *H. glycines*. Also, changes in gene expression have been monitored in *Heterodera glycines-*susceptible soybean cultivar (Kent) using microarray at 6, 12, and 24 hours after infection as well as 2, 4, 6, and 8 days after infection [17]. In that study, the level of genes encoding WRKY6 transcription factor and lipoxygenase were shown to be up-regulated at most time points tested (12 hours after infection (hai) - 8 days after infection (dai)) after infection with *Heterodera glycines*.

Analysis of microarray data can be complex, as datasets are very large and it is difficult to analyze and integrate changes in metabolic pathways. Tremblay *et al. *[18] used the PAICE program to analyze microarray data of soybean leaves infected with soybean rust (*Phakopsora pachyrhizi*). The PAICE program overlays gene expression results from microarrays onto biochemical pathways found in the Kyoto Encyclopedia of Genes and Genomes (KEGG). PAICE makes key changes in gene expression in biochemical pathways stand out and makes comparison of pathway changes among treatments and across time points easier.

New targets for nematode control could be developed through the identification of genes that are involved in the establishment of the nematode in the host plant and which participate in the formation of the permanent feeding site for the nematode. Ibrahim *et al. *[19] were able to control *M. incognita *development in soybean plants after silencing four *M. incognita *genes using the RNA interference mechanism. In this study, portions of the genes encoding mitochondrial stress protein and tyrosine phosphatase were shown to have the greatest effect among four tested genes on nematode development and on the number of galls formed on the RNAi-expressing roots. Also, Dalzell *et al. *[20] were able to silence the gene encoding FMRF amide-like peptide (flp) with 21 bp siRNAs, specific to that gene in infective (J2) stage juveniles of potato cyst nematode, *Globodera pallida*, and *Meloidogyne incognita*. Charlton *e*t *al*. [21] reduced the number of *Meloidogyne incognita *by 50% after simultaneous suppression of two genes, dual oxidase and a subunit of a signal peptidase required for the processing of nematode secreted proteins, respectively.

In this paper we used the 37,500 probe set Affymetrix Soybean GeneChip to assay gene transcript abundance in galls formed in soybean by *M. incognita *at two stages, small galls at 12 dai and large galls at 10 wai. These time points were chosen to contrast active nematode feeding at 12 dai with plant gene expression in a mature infection at 10 wai. The latter time point is particularly interesting as gene expression in plant roots after prolonged infection has not been reported previously. We used PAICE [18] software to visualize the expression of genes related to major biochemical pathways and we identified a number of different pathway genes that were affected by nematode infection. Although we are using biochemical pathways as a format to visualize gene expression results, the changes in gene expression overlaid on these pathways do not imply similar changes in the levels of the encoded protein. This study provides insights into the interaction between *M. incognita *and soybean and into the formation and maintenance of giant cells. Our long-term objective is to identify possible gene targets for manipulation to develop broad resistance of plants to RKN by using gene silencing technology or to over express certain soybean genes.

## Methods

### Plant and nematode procurement

*Glycine max *cv Williams 82 and *M. incognita *population LESREC (Lower Eastern Shore Research and Education Center) were grown in a greenhouse at the United State Department of Agriculture Soybean Genomics and Improvement Laboratory, Beltsville, MD, USA. *M. incognita *eggs were harvested from roots of *G. max *cv Williams 82 2-4 months after inoculation using a method modified from those previously described in Meyer *et al. *[22] and Nitao [23]. Soybean seedlings were grown in Promix (Premier Horticulture INC., Quakertown, PA, USA) for one week in 20 × 20 × 10 cm flats, then moved to sand (The Stone Store, Hanover, MD). Three thousands eggs were used to inoculate roots of 7 day old soybean seedlings (cv. Williams 82). Soybean roots at 12 dai, 10 wai, and control uninfected plants were washed with sterile water, flash frozen in liquid nitrogen, ground to a fine powder and frozen at -80°C until use. The infected roots were collected at 12 days after infection.

Nematodes were stained in infected roots using a modified protocol of Byrd *et al. *[24] and Mahalingam *et al. *[25]. Briefly, roots were washed in gently flowing tap water to remove soil and debris, cut to 2 cm segments, and placed in a small beaker, then soaked in 20-30 ml of 10% commercial Clorox (chlorine bleach, 5.25% NaOC1) for 3 min. The roots were rinsed in tap water and then transferred into a 50 ml glass bottle containing 20 ml of distilled water and left to boil in a microwave with loosened caps. A 500 μl of acid fuschin (0.15 g/10 ml H2O) and 500 μl of glacial acetic acid were added to the root samples and heated to boiling in a microwave twice. The roots were left to cool to room temperature before removing the excess stain with running tap water using Miracloth on the top of the bottle. A 20 ml of clearing reagent (1/3 lactic acid+ 1/3 glycerol + 1/3 distilled water) were added to roots and roots were left to destain for two hours to overnight. The nematodes were stained red as observed in the roots under a dissecting microscope. General chemical reagents were obtained from Sigma Chemical Co (St. Louis, MO).

### RNA extraction and microarray analyses

RNA was extracted from 100 mg each of the three different root samples using the Ultra Clean Plant RNA Isolation Kit (MOBIO, Carlsbad, CA). Gene expression analysis was performed using the GeneChip^® ^Soybean Genome Array (Affymetrix^®^, Santa Clara, CA, USA) containing more than 37,500 probe sets as described in Klink *et a*l. [9,10]. In this GeneChip technology, each high density spot (gene) is represented by 11-probe pairs (11 μm feature size), which allows multiple independent measurement for each transcript. GeneChip^® ^Soybean Genome Array details are available at the Affymetrix^® ^website [26]. The microarrays were hybridized and scanned at the Laboratory of Molecular Technology, SAIC-Frederick, National Cancer Institute at Frederick, Fredrick, MD, USA.

Affymetrix^© ^soybean Genechip data was analyzed as described in Klink *et al. *[9,10,15] with additional analysis using PAICE [18]. Briefly, microarray gene expression data was imported into MATLAB (Natick, MA) Bioinformatics Toolbox. Normalization of the probe sets was performed using RMA (Robust Multiarray Analysis). The resultant calculated output was the log base 2 of the expression values, enabling scaling of the dataset. Volcano plots were produced, which graphically illustrate gene expression fold-change with respect to statistical significance. The plots were produced using fold-changes >= |2.0| and p-values < = 0.05 with respect to the control (untreated roots of cv. Williams 82). The t-test was used in calculating p-values. False Discovery Rate analysis was further utilized against significantly expressed genes [16]. The False Discovery Rate tool 'Significance Analysis of Microarrays' (SAM v3.0; [8] was used with a false discovery rate of 10%.

### RT-PCR for confirmation of differential gene expression in infected soybean roots at 12-dai and 10-wai

To confirm the microarray results conducted obtained for *M. incognita *infected soybean roots at 12-dai and 10-wai, quantitative real time PCR (qRT-PCR) was performed on specific genes that were shown to be differentially expressed during the infection. Fourteen genes were chosen according to the changes in their expression at 12-dai and 10-wai (Table 1). The genes were classified and placed in three different groups according to their function [8,9]; Table 1). Soybean ubiquitin-3 (Accession No. D28123) was used to normalize the results. RNA samples also used for microarray analysis were used in qRT-PCR analysis. RNA from three different biological replicates of each time point (12 dai and 10 wai), and the control (non-infected roots) were used to synthesize first-strand cDNA using the SuperScript First-Strand Synthesis System for RT-PCR (Invitrogen, Carlesbad, CA) following the manufacturer's instructions. Quantitative real time PCR was performed using the Stratagene Mx3000P RT-PCR system as described by the manufacturer (Stratagene, La Jolla, CA) with 10 ng/reaction of cDNA for all genes. Primer sequences specific to each gene are presented in Table 2. Other controls for qRT-PCR included reactions containing no template or no reverse transcriptase. These controls resulted in no amplification. qRT-PCR was performed in two biological replicates and each reaction was replicated three times. DNA accumulation was detected by SYBR Green and the Ct value (cycle at which there is the first clearly detectable increase in the fluorescence) was calculated using the software provided with the Stratagene Mx3000P RT-PCR system. Dissociation curves showed amplification for only one product for each primer set. Data analysis was performed according to the sigmoidal method described by Rutledge and Stewart [27] for absolute quantification of transcripts. Absolute quantification of fluorescence intensity per ng dsDNA was obtained using 100 fg lambda gDNA in quadruplicate to calculate the optical calibration factor. Absolute quantification of the transcript level of the RNAi targeted genes was calculated using specific equations according to Ibrahim *et al. *[19] and Tremblay *et al*. [28].

**Table 1:** List of the 14 induced and suppressed annotated genes in soybean cv. Williams 82 at 12 dai and 10 wai by *M. incognita *(p-value ≤ 0.05) that were used in qRT-PCR for confirmation of microarray results.

Gene name	EC Number	12-dai		10-wai	
		**Accession No**.	**Fold Change**	**Accession No**.	**Fold Change**

Lipoxygenase (LOX1)	1.13.11.12	AW705829	22.6	AW705829	-9.9

Allene oxide synthase (AOS)	4.2.1.92	BG789629	-59.3	BG789629	-77.4

Allene oxide cyclase (AOC)	5.3.99.6	BI943104	-6.3	BI943104	No change

12-oxophytodienoate reductase (OPR2) or (OPR3)	1.3.1.42	BI971360	5.0	CF808146	-8.8

Pectin esterase (PECT.)	3.1.1.11	CF808202	47.5	Not tested	

Cellulase (CELL.)	2.4.1.12	BQ612445	-4.6	No tested	

Caffeoyl-coA-0-methyltransferase (CCOA-OMT)	2.1.1.104	AW349604	2.6	BG155028	1.9

Coumarate 3-hydroxylase (C3H)	1.14.13.-	BU549690	2.9	CD413163	8.4

ferulate-5-hydroxylase (F5H)	1.14.-.-	BU547972	15.4	BU547972	20.5

Cyclin-dependent kinase B2;2 (CDKB2)	No EC	CD418070	5.2	CD418070	3.1

Cyclin-dependent protein kinase (CYCD3)	No EC	AW760424	No change	AW760424	-2.9

**Table 2:** Primers used in RT-PCR reaction to confirm microarray expression data.

Name	Sequences (5'-3')	Product size (bp)
UBI-F	5-GTGTAATGTTGGATGTGTTCCC-3	107
	
UBI-R	5-ACACAATTGAGTTCAACACAAACCG-3	

LOX1-F	5-ATGTTCACCCGTATCATGTTCA-3	100
	
LOX1-R	5-GGGTATCAAATCCAAGTTGGTG-3	

AOS-F	5-CCGCATCCAAAAGTACCAGT-3	244
	
AOS-R	5-TTTGAGGAGGGAGTGTTTGG-3	

AOC-F	5-TGCACGAGCAATTATTGTTGA-3	201
	
AOC-R	5-TTCGGTTTTACACATTAGCATTT-3	

OPR2-F	5-GGTGCATCAACCTCAAACCT-63	218
	
OPR2-R	5-GTTGAGCCAAGATGGGACAT-3	

OPR3-F	5-ACCGGTGCAGGTTCTTAATG-3	176
	
OPR3-R	5-GCCCACTTGTTTCTGCAAAT-3	

PECT-F	5-CAAGAAACCCTCCCAACAAA-3	239
	
PECT-R	5-ACCACTCCATTCCATCCAAC-3	

CELL-F	5-CTGCACAATTCCAGCTGTGT-3	207
	
CELL-R	5-GCGAAAAGGTGAGCAGAAAC-3	

CCOA-OMT (12)-F	5-TTCCATATGTGGCAGGTGAA-3	222
	
CCOA-OMT (12)-R	5-TATGGAATGGGTCCGTGGT-3	

CCOA-OMT (10)-F	5-CGCCTTGGAAATTGGTGTAT-3	232
	
CCOA-OMT (10)-R	5-GAAATCCAACGACCCCTTTT-3	

C3H (12)-F	5-GCAGCCACTGTTTTCCTCTT-3	182
	
C3H (12)-R	5-ACCAGTGATGGGACTCCAAG-3	

C3H (10)-F	5-GCAGGGACAGCGTTGATTAT-3	160
	
C3H (10)-R	5-TATCAGTGGGAGCTGCTGTG-3	

F5H-F	5-AAATGTCATGCCTGGACACA-3	152
	
F5H-R	5-CATGGGCAATTGGAAGAGAT-3	

CDKB2-F	5-CAGCCGTTGACAGACTTTGA-3	203
	
CDKB2-R	5-GGTGGACATTTGGTCCGTAG-3	

CYCD3-F	5-AAATGCGACTGCCTTCTGTC3-	247
	
CYCD3-R	5-ACCAGATCCCTTTTCCACCT-3	

### Pathway Analysis

Biochemical pathway analysis was conducted using PAICE (Pathway Analysis and Integrated Coloring of Experiments) [18]. This software program maps expression levels of genes encoding enzymes found in the KEGG biochemical pathways database. Gene expression levels are denoted using color codes displayed at the pathway nodes depicted by enzyme EC numbers. Besides the pathway mapping feature, PAICE colors EC accessions using gradients of green and red to represent induced and suppressed gene expression respectively. The colors can be tailored to the analysis through the software package.

PAICE has the unique ability to color in yellow the expression of genes having multiple family members that lack a consensus in gene expression, i.e. some members are over-expressed and others are under-expressed.

## Results

### Histological examination of RNK infection

**Figure 1:**
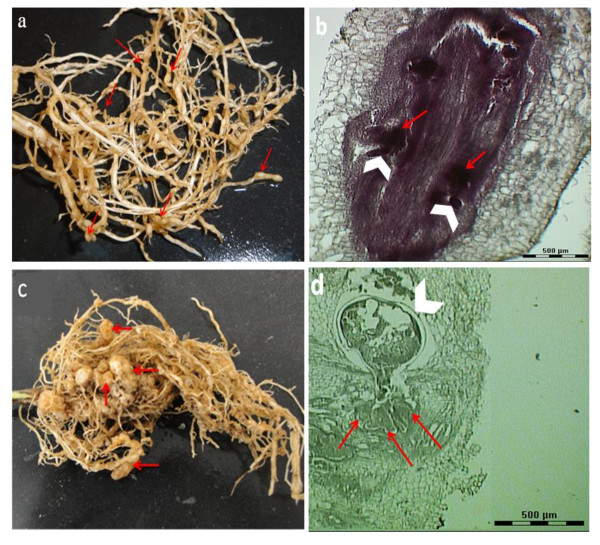
**Soybean roots infected by *M. incognita *at different time points**. (a) galls formed on soybean roots at 12 dai; red arrows point to the galls; (b) giant cells and *M. incognita *in soybean roots at 12 dai (taken by LCM; bar 500 μm); red arrows point to giant cells; white arrowheads points to the nematode; (c) galls formed on soybean roots at 10 wai; red arrows point to the galls; (d) giant cells and *M. incognita *in soybean roots at 10 wai (taken by LCM; bar 500 μm); red arrows point to giant cells; white arrowhead points to the nematode

At 12 dai, galls can be identified as small swellings along the soybean root (Figure 1a). Within the gall the nematode has started feeding and can be visualized by staining with acid fuchsin [24,25] to monitor nematode invasion and development inside the roots (Figure 1b). Mature galls are present on soybean roots at 10 wai (Figure 1c). Within the gall, mature female *M. incognita *can be identified easily by staining (Figure 1d).

### Transcript profiling of galls formed by *M. incognita *infection

**Figure 2:**
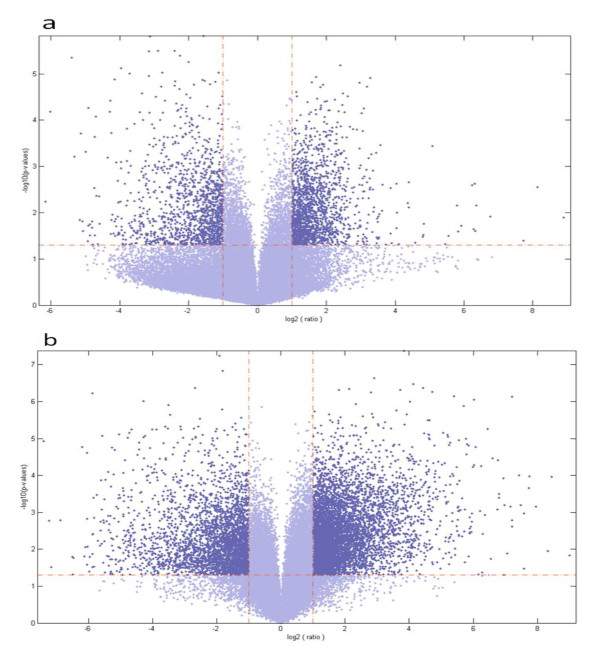
**Volcano blots show significant changes in gene expression for soybean roots after infection with *M. incognita***. (a) Changes in gene expression at 12 dai (b) Changes in gene expression at 10 wai. The fold change was >1.5 with p values < 0.05, determined individually for each gene. The vertical lines on each graph represent p = 0.05. The horizontal lines represent the log2 of gene expression.

A comparison of gene expression at12 dai compared to control led to the identification of 1867 genes with greater than 1.5-fold change in expression (Figure 2). Of these, 1278 genes increased and 589 genes decreased in expression. Transcripts encoding leghemaglobin C1 (AI973819) increased the most at 386-fold. The most down-regulated gene was BF070134 with homology to a putative senescence protein 12 and to ERD7; its transcripts were 77-fold lower than in the control. There were 2108 genes with altered expression in galls at 10 wai. Of these, 1460 genes increased in expression and 648 genes decreased in expression. The transcript of the gene encoding pathogenesis related protein PR1a (CK605838) increased the most at 258-fold. As in the 12 dai experiment, the most down-regulated gene was BF070134 with transcripts 172-fold lower than the control. When gene expression at 10 wai was compared directly to 12 dai, 827 genes were up-regulated, while 535 genes were down-regulated. In this case, transcripts of the gene encoding the cysteine-rich plant defense protein, defensin (PDF2.3; BI321308), increased the most at 63-fold, while the transcripts of the gene encoding xylene serine peptidase 1, subtilase (AW309540) decreased the most at 126-fold.

### Mitosis and cell division

Our data reflect changes in expression of numerous genes involved in nuclear regulation and cell division in the gall at 12 dai and 10 wai (Table 3). For example two genes were increased in transcript abundance that are regulators of the cell cycle. These genes encode two NDR (nuclear Dbf2-related) family members of AGC kinase, and they are increased in expression 24.5-fold (BI968028) and 5-fold (CA802074)) at 12 dai. By 10 wai genes of several NDR family members (NHL1 and NHL3) are expressed less than at 12 dai, i.e. BI968028 at 5.5 fold, AW156706 at -2.6 fold, and CF806406 at -9.6 fold. Transcripts of numerous cyclin-dependent protein kinases are in greater abundance at 12 dai than in control tissues (Table 3). This correlates well with the increase in nuclear division that occurs in giant cell. In addition, the gene encoding RBR1 retinoblastoma-related protein, which modulates E2F-transcripton factors that inhibit cell proliferation, is also increased at 12 dai. Expression of a gene encoding the plant hormone, phytosulfokine 3 growth factor (BK000117) involved in positive regulation of plant cell proliferation, is increased by 44-fold at 10 wai.

**Table 3:** Cyclin dependent kinases genes that were greatly induced and suppressed in soybean cv. Williams 82 at 12 dai and 10 dai by *M. incognita *(p-value ≤ 0.05).

Probe set	**Genbank accession No**.	Gene annotation	Fold change at 12 dai	Fold change at 10 wai
Gma.2284.1.S1_at	CD418070	CDKB2;2	5.202209	3.123

Gma.2284.2.A1_at	CD395891	CDKB2;	4.586837	2.466153

Gma.7205.1.A1_at	AW756085	CYCD3;2	No changes	-5.821128

Gma.17909.1.S1_at	AW760424	CYCD3;2	No changes	-2.853675

Gma.5497.1.S1_at	CD414002	CKS1 (CDK-SUBUNIT 1);	No changes	2.003443

Gma.5497.1.S1_s_at	CD414002	CKS1	No changes	1.884626

Gma.17909.1.S1_at	BU546339	CYCP1;1	No changes	-2.933661

### Cell wall modifications

**Figure 3:**
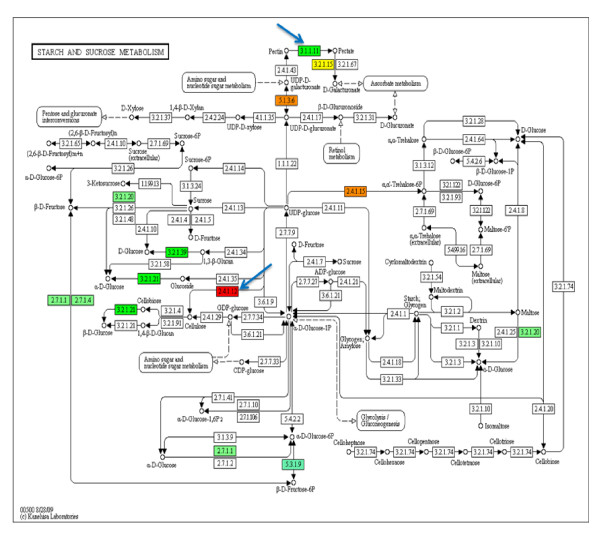
**Transcript abundance of genes encoding enzymes in the pathway of starch and sucrose metabolism as represented by KEGG**. Relative transcript abundance of genes encoding enzymes in the pathway at 12 dai compared to uninfected control overlaid as colors in the boxes containing the enzyme commission (EC) number of the enzyme. Enzymes colored in green are encoded by up-regulated genes. Boxes colored in red represent down-regulated genes. Boxes colored in yellow represent a gene family and different gene members are up- and down-regulated, respectively. The blue arrows point to pectin esterase [EC: 3.1.1.11] and cellulose synthase [EC: 2.41.12]. The blue arrows point to pectin esterase [EC: 3.1.1.11] and cellulose synthase [EC: 2.41.12].

Numerous genes involved in cell wall remodeling were shown to be differentially expressed after infection with *M. incognita *(Table 4). Genes encoding four members of the expansin enzyme family (expansins A1, A5, A16, and 45) were up-regulated at both time points (Table 4). Also, genes encoding endo-1,3-beta-glucanase [EC 3.2.1.60] family members were differentially expressed (Table 4). Most of them were up-regulated, while one member (AW831345) was down-regulated 27-fold in the 12 dai and 44-fold in the 10 wai. A gene encoding the cell wall-modifying xyloglucan endotransglycosylase/hydrolase [EC 2.4.1.207] was down-regulated at both time points, while the gene encoding endoxyloglucan transferase A2 was up-regulated at both time points (Table 4). Expression of a gene encoding pectin esterase [EC 3.1.1.11] increased 24-fold and 47.5-fold at 12 dai and 12 wai, respectively. In addition, a gene encoding cellulose synthase [EC 2.41.12] was down-regulated by 9.3-fold and 4.5-fold at 12 dai and 10 wai, respectively (Figure 3).

**Table 4:** Enzymes involved in cell wall modification and remodeling, for which their respective RNAs were found to be differentially regulated in our microarray dataset at both time points (12 dai and 10 wai; p-value ≤ 0.05).

Probe set	**Genbank accession No**.	12 dai_Fold Change	10 wai_Fold Change	Gene Family
Gma.13110.1.S1_at	CD394837	24.68157	63.82636	Expansin A1

Gma.7784.1.A1_at	BM269985	9.148853	14.15569	Expansin A1

GmaAffx.90097.1.S1_at	CF805822	6.665491	7.075862	Expansin A16

GmaAffx.67477.2.S1_at	AW509184	6.36759	3.798536	Expansin A5

Gma.14216.1.A1_at	CD417846	No change	1.854507	Expansin 45

GmaAffx.86629.2.S1_at	BQ742947	10.92815	33.72573	Endo-1,3-beta-glucanase

Gma.6574.1.S1_at	BQ628332	5.983501	4.524413	Beta-1,3-glucanase

GmaAffx.71901.1.S1_s_at	BE022356	No change	4.844002	Beta-1,3-glucanase

Gma.2801.1.S1_at	BI785739	No change	4.21076	Beta-glucosidase

GmaAffx.91055.1.S1_at	CF806780	4.618147	3.953466	Beta-1,3-glucanase

Gma.17299.2.S1_at	BG237465	No change	3.947161	Beta-1,3-glucanase-like protein

GmaAffx.87027.1.S1_at	AW831345	-27.19943	-43.94289	Beta-1,3-glucanase 2

GmaAffx.89396.1.S1_x_at	CK605572	-6.914317	-9.101676	Xyloglucan

GmaAffx.34697.1.S1_at	AW433263	-16.99092	-30.42019	Endoxyloglucan transferase A4

**Figure 4:**
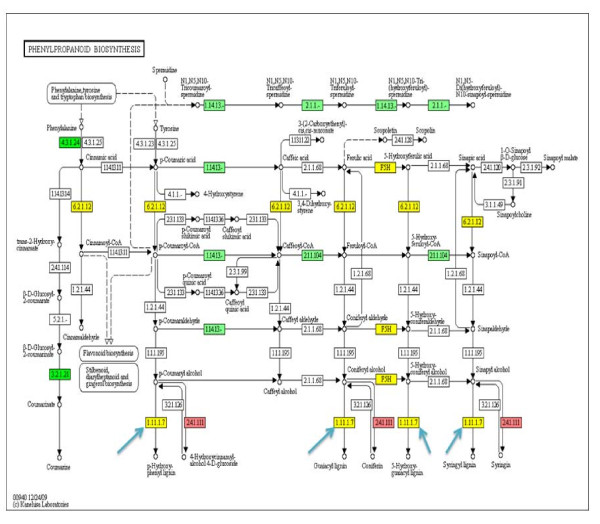
**Expression profiles of the genes encoding enzymes in phenylpropanoid biosynthesis pathway at 12 dai**. The blue arrows point to extensin peroxidases [EC: 1.11.1.7] that participate in lignin biosynthesis. Boxes colored in red represent down-regulated genes encoding that enzyme. Boxes colored in green represent up-regulated genes. Boxes colored in yellow represent multiple genes encode the enzyme and some of those different gene copies are up-regulated, while others are down-regulated.

Also, in phenylpropanoid biosynthesis, genes encoding a family of 21 extensin peroxidases [EC 1.11.1.7] that participate in lignin biosynthesis were differentially regulated (Figure 4). The extensin gene with the highest increase in expression (AW309606) increased by 95-fold while the extension gene with the largest decrease in expression (BI970840) decreased by -16-fold.

### Carbon and energy metabolism

**Figure 5:**
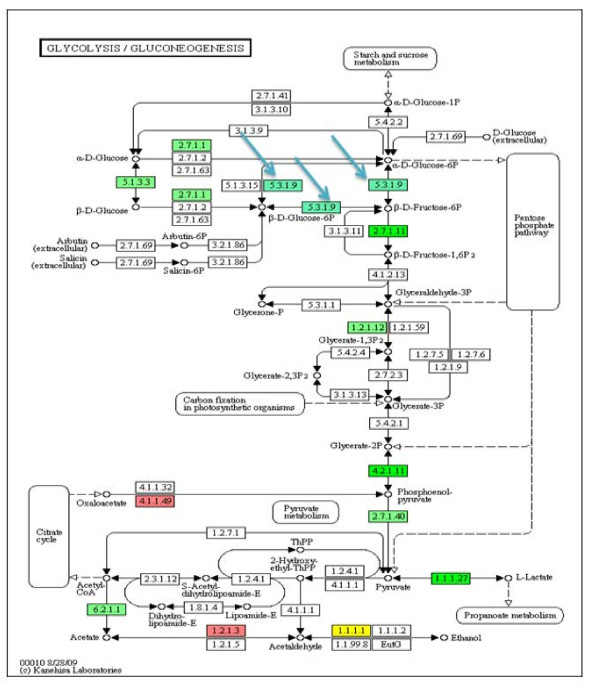
**Expression profiles of the RNAs encoding enzymes in glycolysis/gluconeogenesis pathway**. The blue arrows point to glucose-6-phosphate isomerase [EC: 5.3.19]. Boxes colored in red represent down-regulated genes encoding that enzyme. Boxes colored in green represent up-regulated genes. Boxes colored in yellow represent multiple genes encode the enzyme and some of those different gene copies are up-regulated, while others are down-regulated.

The expression of numerous genes encoding enzymes in glycolysis, the tricarboxylic acid cycle and in amino sugar synthesis was altered. For example, the gene encoding UDP-glucuronate 4-epimerase [EC 5.1.3.6] was greatly down-regulated with a 21-fold decrease in transcript abundance. Also, the gene encoding GDP mannose 4,6-dehydratase [EC 4.2.1.47] was down-regulated 20.5- and 5.3-fold at 12 dai and 10 wai (data not shown). In the glycolysis pathway, many genes were up-regulated during infection, including genes encoding glucose-6-phosphate isomerase [EC 5.3.1.9], transcripts of which were increased by 14-fold at 12 dai (Figure 5)

### Defense-related genes

**Figure 6:**
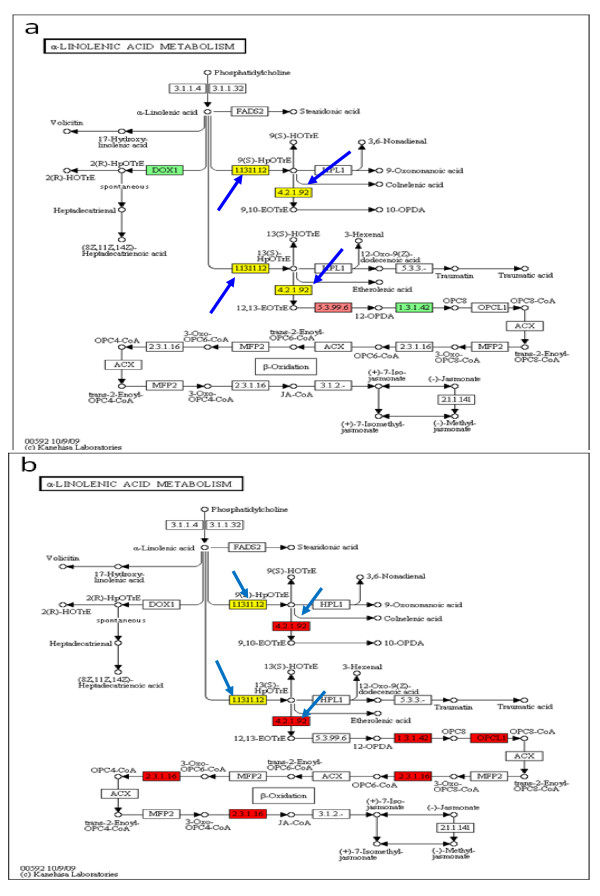
**Expression profiles of the RNAs encoding enzymes in the alpha-linolenic acid pathway**. (a) 12 dai; (b) 10 wai. The blue arrows point to lipoxygenase [EC: 1.13.11.12] and allene oxide synthase [EC: 4.2.1.92]. Boxes colored in red represent down-regulated genes encoding that enzyme. Boxes colored in green represent up-regulated genes. Boxes colored in yellow represent multiple genes encode the enzyme and some of those different gene copies are up-regulated, while others are down-regulated.

There are multiple changes in expression of genes encoding enzymes of the alpha-linolenic acid pathway leading to several important defense-related compounds, including jasmonic acid (Figure 6). At 12 dai, the change in expression of genes encoding enzymes leading to jasmonic acid seems conflicting. For example, the gene encoding palmitoyl-CoA hydrolase [EC 3.1.2.2], an enzyme that is needed for the biosynthesis of the alpha-linolenic acid, is suppressed. Linolenic acid can be a substrate for lipoxygenase [EC 1.13.11.12]. Genes encoding six lipoxygenase family members were differentially expressed. Mostly of them were up-regulated with highest induction of 22-fold for one member (AW705829). One gene family member (BI426746) was down-regulated by 3.8-fold. In addition, some gene family members encoding allene oxide synthase (hydroperoxide dehydratase [EC 4.2.1.92]), leading to jasmonic acid synthesis, were up-regulated, while others were down-regulated. The abundance of transcripts of the gene encoding the next enzyme in the pathway, allene oxide cyclase [EC 5.3.99.6] was also decreased. Yet, a gene encoding OPDA reductase [EC 1.3.1.42] was up-regulated. In contrast, most of the genes encoding enzymes involve in jasmonic acid biosynthesis were down-regulated at 10 wai.

### Genes encoding transcription factors and other proteins

Changes in gene transcripts were accompanied by changes in expression of transcription factors, especially those in the WRKY family of transcription factors. Our microarray results indicated that genes encoding several family members of WRKY genes were down-regulated at 12 dai, including genes encoding WRKY6, 15, and 22 (-10.5, -14.3, and -8.5 FC, respectively). In contrast, at 10 wai, genes encoding WRKY 21 and 70 were up-regulated at 117 and 42 FC, respectively.

Several pathogenesis-related proteins are induced in plants during infection with any pathogen or by wounding, including nematode infection, and induction of many of these is affected by salicylic acid, jasmonic acid or ethylene [29]. In our microarray data, genes encoding pathogen related proteins (PR) such as PR3 were down-regulated at 12 dai and genes encoding PR3 at 10 wai showed a mixed response; some were up-regulated while others were down-regulated. The three copies of the pathogen related protein PR1 gene were over-expressed by 78.23, 97.56, and 138.50 fold, respectively (Data not shown).

### Confirmation of differential gene expression by quantitative PCR

**Figure 7:**
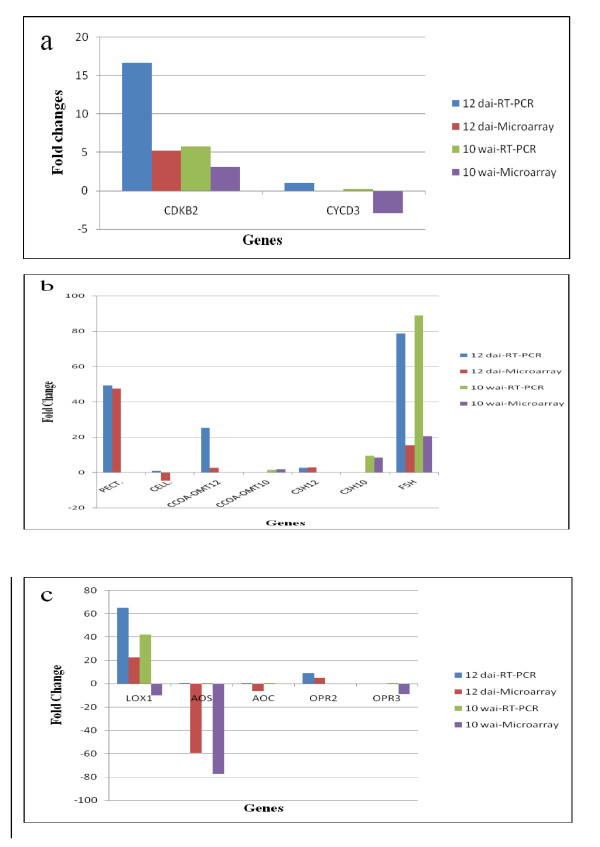
**Microarray results were validated by comparison of microarray fold-change with qRT-PCR for specific genes**. (a) genes involved in cell cycle progression; (b) Genes involved in cell wall remolding and modification; (c) genes participating in jasmonic acid biosynthesis.

Quantitative PCR was conducted to confirm gene expression patterns revealed by microarray analysis. We measured transcript abundance of 14 genes that showed increased or decreased transcript abundance by microarray analysis (Table 5; Figure 7). The trends in up- or down-regulation of gene transcripts were consistent between microarrays and quantitative PCR results except for expression of the gene encoding lipoxygenase family member LOX1 at 10 wai. However, we did observe differences in levels of expression between methods. Differences in fold-change in gene expression as measured by microarray and qRT-PCR have been reported in previous studies [15,18,28].

**Table 5:** Comparison of microarray and quantitative real-time-PCR (qRT-PCR) quantifications of gene expression.

Gene	Fold Change comparison for 12 dai		Fold Change comparison for 10 wai	
**Jasmonic acid**	RT-PCR	Microarray	RT-PCR	Microarray

LOX1	65.3	22.6	42.2	-9.9

AOS	0.0	-59.3	0.0	-77.4

AOC	0.1	-6.3	0.3	0

OPR2	9.1	5		

OPR3			0.1	-8.8

**Cell Wall**				
				
PECT.	49.4	47.5		
		
CELL.	1.0	-4.6		
		
CCOA-OMT12	25.0	2.6		

CCOA-OMT10			1.5	1.9

C3H12	2.7	2.9		

C3H10	0	0	9.4	8.4

F5H	78.6	15.4	88.9	20.5

**Cell Cycle**				
				
CDKB2	16.7	5.2	5.8	3.1

CYCD3	1.0	0	0.2	-2.9

Note: Gray boxes refer to untested genes in a specific time point.

## Discussion

When *M. incognita *infects and feeds in a soybean root, numerous genes are altered in expression in the root. *M. incognita *not only triggers the defense response of the root, but also redesigns the morphology of the root to form a gall and converts a soybean cell into a giant cell for feeding. The timing of these changes coincides with changes in gene expression as seen in our microarray experiments.

### Regulators of the cell cycle and cell division

The cell cycle is regulated by two types of cyclin-dependent kinases. CDKA is required for cells to enter the S and M phases. CDKB1 and CDKB2 are expressed during the G2 and M phases and are responsible for the G2-M transition [30]. Our microarray results indicate that genes encoding some members of the cyclin-dependent kinases family were differentially expressed at 12 dai and 10 wai. Over-expression of the gene encoding CKB2 at 12 dai (5.2 and 4.6 FC) correlates with the increase in plant nuclear division that occurs at the infection site due to *M. incognita *infection and feeding. Cells selected by *M. incognita *for feeding become multinucleate giant cells. This increase in nuclear division continues until 14 dai [3-5].

Cell cycle activation in giant cells has also been observed by Engler *et al. *[31]. In that study, the transition from S to G2 and G2 to M phase was reported after the over-expression of a GUS gene driven by the cycB2 or cycA2 promoters at one to nine days after infection with *M. incognita*. Expression of the CDKB2 gene at 12 dai was higher than at 10 wai, i.e., 5.2- versus 3.1-fold, respectively (Table 1). Ramsay *et al*. [32] found that cyc D3 is essential to stimulate the G1 phase of the cell cycle in root knot nematode infected giant cells. In this investigation, the two types of CycD3 (CycD3.2 and CycD3.3) were shown to be relatively more strongly expressed as compared to that of LeCycA1.1, LeCycB1.1 and LeCycD3.1 in giant cells induced by *Meloidogyne spp*. compared with other cyclin-dependent kinases. They observed PCR amplification of CyD3.2 and CycD3.3, while no amplification of cycA.1, CycB1.1 and CycD3.1 was observed. Our data showed a suppression of gene expression of the gene encoding cycD3 which is important for the regulation of the G1-S transition. In addition, at 10 wai we found an increase in gene expression of CKS1 (CDK-Subunit 1), a protein that prevents CDK from driving the cell cycle into S phase. This result suggests that at the earlier time point, the giant cells reach maturity and then the genes required for nuclear division are turned off.

### Cell wall modification and remodeling

Due to multiple nuclear divisions of selected cells with no coincident cell division, the giant cells sometimes reach more than 400-times the size of a normal cell and may contain more than one hundred nuclei [4]. This expansion of the giant cell is accompanied by extensive cell wall modification. Our microarray data indicate altered expression of many genes involved in cell wall extension and remodeling (Table 4). We found that the genes encoding a cell wall-modifying xyloglucan endotransglycosylase/hydrolase [EC 2.4.1.207] and endoxyloglucan transferase A2 are differentially expressed in soybean roots after infection with *M. incognita *(12 dai and 10 wai). These enzymes play a role in softening and breaking down the cell wall [33]. Genes encoding many endo-1,4-glucanase family members were up-regulated at both time points (12 dai and 10 wai). Endo-1,4-glucanase is involved in cell wall remodeling and expansion. LCM was used to isolate giant cells formed in tomato by *M. javanica *to examine gene expression [34]. Numerous transcripts of genes involved in cell wall remodeling were also identified in the cDNA library of giant cells 4 dai, including transcripts of genes encoding pectin methylesterase and pectinesterase. Goellner *et al*. [35] identified genes encoding endo-1,4-glucanases that were up-regulated in feeding cells formed by *M. incognita *and cyst nematode in tobacco plants. Also, Mitchum *et al. *[36] found that the promoter of an endo-1,4-β-glucanase gene was strongly activated in feeding cells formed by *Meloidogyne incognita *as indicated by strong promoter-driven GUS expression.

The increase in expression of the gene encoding expansin A in our results is consistent with other investigations, wherein the expansin (LeEXPA5) genes in *A. thaliana *and tomato were shown to be up-regulated in developing giant cells after infection with *Meloidogyne *[37,38]. Moreover, down-regulation of genes encoding cellulose synthase and over-expression of genes encoding pectin esterase that degrades pectin to pectate coincide with a breakdown of the cell wall during the early time points of infection with *Meloidogyne*. Our results are consistent with those of Jammes *et al. *[38], who found genes encoding pectin esterases and pectate lyases were activated in *Arabidopsis thaliana *roots after infection with *Meloidogyne incognita *and the cell wall loosening process was activated during the development of the giant cell as well. In giant cells formed in tomato by *M. javanica*, there is an 8-fold and 7.3-fold increase in expression of the gene encoding pectinesterase U1 precursor [34]. Giant cells formed by *M. javanica *in roots of *Arabidopsis *were collected by LCM and analyzed by Barcala *et al*. [39]. Genes encoding cellulose synthase, expansin, pectate lyase, endoxyloglucan transferase also were all up-regulated in these cells coinciding with cytoskeleton rearrangements that occur during giant cell development.

### Nutrients supply for *M. incognita*

The nematode uses a large amount of plant resources to develop and reproduce. This demand for energy and carbon is reflected in the numerous genes involved in glycolysis and gluconeogenesis that are up-regulated in the soybean root (Figure 5). For example, we found many genes encoding enzymes in the glycolysis pathway and amino sugar synthesis to be up-regulated. Mostly, the changes in gene expression occurred early in infection (12 dai). In addition to their roles in pathways that provide energy and carbon for the nematode, some of these genes have an essential role in the soybean-*M. incognita *interaction. For example, at 12 dai, the gene encoding UDP-glucuronate 4-epimerase (EC 5.1.3.6) is highly down-regulated (-21 fold). In Arabidopsis, a mutation in this gene resulted in hyper-sensitivity to the cyst nematode, *Heterodera schachtii *[40]. In *Azospirillum brasilense*, this enzyme is important for lipopolysaccharide synthesis which is important in the bacterium-plant root interaction. A mutation in this gene resulted in the failure of the bacteria to respond to several stresses and antimicrobial compounds. It also affected the ability of the bacteria to form biofilms [41]. This enzyme may be important in allowing the host to respond to *M. incognita *invasion [42,43].

In the glycolysis and gluconeogenesis pathways, many genes were shown to be up-regulated, including the gene encoding glucose-6-phosphate isomerase [EC: 5.3.1.9]. The gene encoding this enzyme was also shown to be up-regulated in cucumber plants after treatment with *Trichoderma asperrellum *T34 [44]. The enzyme is essential in salinity tolerance in the alga *Dunaliella salina *[45].

Not only do nematodes require large quantities of carbon and energy from its host, they also use starch during juvenile development. Starch is stored in syncytia formed by *Heterodera schachtii *in roots of *Arabidopsis *[46]. The authors postulate that the starch is also used to compensate for fluctuating levels of sugar during the course of nematode development and feeding. The high metabolic rate of cells was suggested by the increased expression of ribosomal genes in giant cells induced by *M. javanica *in tomato roots [47].

Degradation of the cell walls could result in release of sugar which in turn will be channeled to glycolysis as reflected in the activation of the genes encoding enzymes in the glycolytic pathway. Also, since some enzymes in glycolysis participate in the biosynthesis of pentose, purines and pyrimidines, there could be an increase in production of nucleotides required for DNA replication.

### Plant defense system

When a nematode invades a plant root, it must repress or control the plant defense response so it can successfully establish its permanent feeding site [4-6]. Our microarray data showed significant changes in expression of genes related to the defense response against pathogens. The pathway leading to jasmonic acid biosynthesis is one of the pathways associated with pathogen resistance and genes in this pathway were significantly affected by *Meloidogyne incognita *infestation at both time points (12 dai and 10 wai; Figure 6). At 12 dai, six of seven members of the lipoxygenase gene family were up-regulated. Lipoxygenase (LOX) is essential to oxylipin biosynthesis and has an important function in the plant defense response against wounding and pathogen attack [48]. Reduction of LOX activity resulted in an increase in susceptibility of transgenic potato plants to insect attack [49]. Over-expression of the gene encoding lipoxygenase could mean that more 9-HPOTrE would be produced. This is one of the major products of lipoxygenase (Figure 6). Interestingly, 9-HPOTrE is involved in the activation of the plant defense response directly or through its metabolites. In potato plants, 9-HPOTrE is produced in response to injury or infection. The role of 9-HPOTrE in the plant defense response suggests that there may be another pathway of LOX-mediated defense response [-50]. 9-HPOTrE could also be a substrate for allene oxide synthase (Figure 6) to produce OPDA, the precursor for jasmonic acid. At 10 wai, the abundance of the lipoxygenase transcript was much lower than the 12 dai time point. Three of seven gene family members encoding lipoxygenase were down-regulated. Also, all of the allene oxide synthase family members were greatly down-regulated in addition to some other genes encoding enzymes in the jasmonic acid biosynthetic pathway (Figure 6). This indicates that at 12 dai the plant defense system is still struggling to fight the infestation, but after prolonged infection (10 wai) most of the genes that are responsible for the production of one major defense hormone, jasmonic acid, were turned off. Genes in this pathway could be targets for testing whether resistance to nematode infestation can be increased in transformed plants by over-expression of these genes.

We found a number of genes encoding PR proteins that were differentially expressed in soybean roots 12 dai with *M. incognita*. The genes encoding PR-1, PR-2 and PR-5 protein families increased in expression. Genes encoding PR-2 and PR-5 are expressed in *sid *mutants of *Arabidopsis *that do not accumulate SA. However, genes encoding PR1 are known to be induced by salicylic acid (SA) [50,51]. This suggests that salicylic acid or its derivatives may be synthesized at 12 dai and 10 wai by *M. incognita *infection. Interestingly, there are two different possible routes to salicylic acid production [52]. The pathway that has the most scientific support involves isochorismate synthase [53] and its genes are not represented on the microarray. The other pathway leading to SA production involves phenylalanine. In this pathway, we found a large increase in the expression of the gene encoding tyrosine aminotransferase [TAT; EC:2.6.1.5]. If the abundance of this enzyme is increased, then this could lead to increased phenylalanine production. Genes encoding phenylalanine ammonia-lyase [EC:4.3.1.24]; and salicylate 1-monooxygenase [1.14.13.-] were over-expressed 6.9 and 2.9 FC, respectively. SA induces the expression of PR-1 [54]. In giant cells of tomato formed by *M. incognita *4dai, several genes involved in the phenylpropanoid pathway were detected, notably phenylalanine ammonia-lyase and cinnamyl alcohol dehydrogenase (35). Transgenic tobacco over-expressing PR-1 was more resistant to blue mold and black shank caused by *Peronospora tabacina *and *Phytophthora parasitica f. sp. nicotianae*, respectively [29]. Although SA treatment of tomato plants that were inoculated with *Meloidogyne incognita *did not completely eliminate nematode infection, it enhanced the synthesis of PR-1, which resulted in a significant increase in resistance to the nematode [55]. In contrast, Portillo *et al*. [56] reported a decrease in transcripts of the PR-1 precursor in giant cells collected from tomato infected with *M. javanica *by LCM as measured using qRT-PCR. Genes encoding PR-3 and PR-4 family proteins are reported to be up-regulated by jasmonic acid and ethylene [29]. Also, PR-4 showed ribonuclease activity against fungal protein in wheat [29]. Furthermore, a gene encoding a pathogenesis-related protein was reported as up-regulated in the interaction of *M. incognita *with *Arabidopsis *at 21 dai [57]. They also reported the increased expression of genes encoding several proteinase inhibitor proteins and leucine-rich repeat family proteins.

## Conclusion

There are major changes in host gene expression between 12 dai and 10 wai by *M. incognita*. In the pathway leading to jasmonic acid synthesis, several genes are down-regulated at 10 wai. We identified changes in important genes and pathways during parasitism. Some host genes encode proteins that participate in the establishment of the feeding site, i.e., giant cells, required by *M. incognita *and for gall formation. These results provide new insights into host-parasite interactions. In the future, some of these genes may be used to control the plant parasitic nematode infestation through plant genetic engineering to over-express defense genes or silence genes that promote giant cell and gall formation.

## Abbreviations

RKN: root-knot nematode; SCN: soybean cyst nematode; PCR: polymerase chain reaction; Dai: day after infection; LCM: laser capture microdissection; NDR: nuclear Dbf2-related; FC: fold change; Wai: week after infection; KEGG: Kyoto encyclopedia of genes and genomes; JA: jasmonic acid; SA: salicylic acid

## Authors' contributions

HMMI, designed the study, managed plants and nematodes, isolated samples, analyzed data, performed qRT-PCR, wrote manuscript, revised manuscript; PH, performed microarray analyses, revised manuscript; NWA, performed statistical analyses of microarray analyses, revised manuscript; EHAH, coordinated and helped to draft the manuscript, critical reviewed manuscript; AEYGED, coordinated and helped to draft the manuscript, critical reviewed manuscript; MAMA, designed the study, critical reviewed manuscript; BFM, designed the study, analyzed data, contributed to writing and editing of the manuscript.
